# Supplementary material for: Vinblastine/Methotrexate for Debilitating and Progressive Plexiform Neurofibroma in Children and Young Adults with Neurofibromatosis Type 1: A Phase 2 Study
Source: Cancers (Basel). 2023 May 5;15(9):2621. doi: 10.3390/cancers15092621 (PMC10177272; doi:10.3390/cancers15092621)
Supplement: Supplementary file 1 [file cancers-15-02621-s001.zip › cancers-2330094-supplementary.pdf]

Table S1. Comparison of tumor status at end of therapy by 2-dimensional and 3-dimensional imaging analyses, by participant.

End of Treatment Imaging Outcomes (N=15)		3-Dimensional MRI	
		Progressive Disease	Stable Disease
2-Dimensional MRI	Progressive Disease	2	0
	Stable Disease	5	8

Abbreviations: N, number; MRI, magnetic resonance imaging
